# Gene–Environment Interaction in the Era of Precision Medicine – Filling the Potholes Rather Than Starting to Build a New Road

**DOI:** 10.3389/fgene.2020.00921

**Published:** 2020-10-06

**Authors:** José M. Álvarez-Castro

**Affiliations:** Department of Education, University and Professional Training, Xunta de Galicia, Santiago de Compostela, Spain

**Keywords:** gene–environment interaction, gene–environment correlation, precision medicine, disease susceptibility, COVID-19, mathematical model, NOIA

## Abstract

Gene–environment interaction is a key part of evolutionary biology, animal, and plant breeding, and a number of health sciences, like epidemiology and precision medicine. However, bottlenecks in models of gene–environment interaction have recently been made manifest, particularly in the field of medicine and, consequently, specific improvements have been explicitly requested—namely, an implementation of gene–environment interaction satisfactorily disentangled from gene–environment correlation. The present paper meets those demands by providing mathematical developments that implement classical models of genetic effects and bring them up to date with the prospects current available data bestow. These developments are shown to overcome the limitations of previous proposals through the analysis of illustrative examples on disease susceptibility, with special attention paid to precision medicine. Indeed, a number of misconceptions about the application of models of genetic/environmental effects to precision medicine are here identified and clarified. The theory here provided is argued to strengthen, in particular, the methodology required for high-precision characterization of strain virulence in the study of the COVID-19 pandemic.

## Introduction

Scientific progress is often accompanied with expectations beyond objective appraisal. On the one hand, quantitative trait locus experiments became prominent 30 years ago and substantial resources were soon after expectantly invested for elucidating genetic architectures of traits of economic importance (see e.g., Rifkin, 2012; Álvarez-Castro, 2016). In turn, the latest decade witnessed major efforts to aid livestock production and plant breeding to undergo a swift switch toward genomic prediction (see e.g., Gondro et al., 2013). On the other hand, although initially developed for model species, genetic mapping of human traits became possible at the beginning of the current century by means of The International HapMap Project (International_Hapmap_Consortium., 2003) and genome-wide association studies (GWAS; see e.g., Gondro et al., 2013) but—in line with the fate of quantitative trait locus experiments—its potential for dissecting the genetic basis of diseases is openly questioned nowadays (see e.g., Teperino, 2020a).

The aforementioned advances in genetics methodologies have enabled increasingly accurate medical predictions, particularly in regards to treatment efficiencies and prevention strategies for different (groups of) individuals, an approach that has been coined as precision medicine. In this context, the first half of the title above, “Gene–environment interaction in the era of precision medicine,” has been stolen from a recent paper in which bottlenecks of classical models of genetic effects and their use in genetic mapping are discussed (Li et al., 2019). This is so because the present paper takes the baton of the aforementioned one by reviewing whether it is realistic and worth it to try and further amend classical models of genetic effects or whether it proves more sensible (or even necessary) to undertake alternative theoretical strategies instead.

In order to further feed into that debate, the present paper dissects the advantages and limitations of the current theory of gene–environment interaction stemming from the classical models of genetic effects and provides a new mathematical implementation that overcomes their historical limitations. Next, the advantages of the theory here provided are shown through built-in cases on disease susceptibility, which also serve to further illustrate the application of this theory to make predictions in the aid of precision medicine. Then, in the discussion, all the above is argued to endorse both the general flexibility of the classical models of genetic effects to serve as a basis for further implementations and, particularly, the theory here provided to enable a demanded leap in the application of gene–environment interaction for medical purposes, like the achievement of a detailed understanding of important facts of the current COVID-19 pandemic.

## Previous Models of Gene–Environment Interaction

Firstly, the basic conceptual definition of gene–environment interaction is here discussed. The genetic and environmental components act independently as long as environmental changes cause the same effects on phenotypes for all genotypes. If, for instance, genotype G_1_ displays phenotypes 1 and 3 under environments E1 and E2, respectively (i.e., the environmental change causes an increase of two phenotypic units to this genotype), and genotype G_2_ displays a phenotype of 2 under environment E_1_, then it is said that there is gene–environment interaction whenever genotype G_2_ displays a phenotype different from 4 under environment E_2_.

### An Alternative Road Planned for Modeling Gene–Environment Interaction

In the aforementioned paper, Li et al. (2019) echo the message that techniques using conventional genetic models do not often provide insightful enough results and that, in particular, they have so far provided no clear-cut evidence on whether disease etiologies are due to rare alleles with strong effects or to common alleles with weak effects. More to the point, Li et al. (2019) have carried out a simulation by means of which certain genetic models are shown not to be able to capture the complexity of realistic underlying factors of a disease—particularly, involving epistatic effects (gene interactions, i.e., departures from the sum of the marginal contributions of the effects of the genes involved).

Further on, Li et al. (2019) provide a probabilistic approach based on a Bayesian framework to hierarchically model gene–environment interaction, leading to a population-dependent index, C, called the genetic coefficient of the disease (at a population)—“a large C indicates large distinguishability of case genomes from control genomes.” Then they illustrate the performance of the proposed methodology using a built-up example in which the disease susceptibility is by default very low (0.01) and it significantly increases due to either environmental (exposure) or genetic (risk allele) factors or both, to 0.4, 0.5, and 0.9, respectively. That case is hereafter referred to as the risk and exposure (RAE) case (see Table 1). With an exposure frequency of 0.24 and a frequency of the risk allele of 0.15, Li et al. (2019) report the genetic coefficient of the disease of the RAE case to be C = 0.79.

**TABLE 1:** Phenotypes (disease susceptibility) of the four individual classes (risk allele carriers and non-carriers under exposed and non-exposed environments) for the two cases considered in the text—the case taken from Li et al. (2019), here called the risk and exposure (RAE) case and the genetic risk to exposure (RTE) case.

		**Genetics**	
**Case**	**Environment**	**Default**	**Risk**
RAE	Default	0.01	0.5
	Exposed	0.4	0.9
RTE	Default	0.01	0.01
	Exposed	0.4	0.9

*Complete dominance of the risk allele is assumed so that homozygotes for the risk allele and heterozygotes are equally susceptible to the disease.*

### The Classical Road-Network of Gene–Environment Interaction

About half a dozen years earlier, Ma et al. (2012) provided a model of gene–environment interaction based on the natural and orthogonal interactions (NOIA) model of genetic effects (Álvarez-Castro and Carlborg, 2007), stemming from the classical models. In these models, the parameter 2α can be used to reflect the “difference between the additive expectations of case genomes and control genomes,” thus providing an alternative measure for the genetic coefficient of the disease, C from the work of Li et al. (2019). Assuming Hardy–Weinberg proportions at the risk allele locus and an equal risk of heterozygotes and homozygotes for the risk allele (since it is not explicitly specified otherwise in that paper), the model from Ma et al. (2012) can be used to compute a difference between the additive expectations of case genomes and control genomes of 2α = 0.85 (or, to be more precise, 2α_G_ = 0.85, using the specific notation from Ma et al. (2012)). The departure between this value and the genetic coefficient of the disease, C = 0.79, from the work of Li et al. (2019), could be due to the choices necessary in relation with dominance and the Hardy–Weinberg proportions.

Along with the aforementioned statistical formulation of genetic effects, both NOIA (Álvarez-Castro and Carlborg, 2007) and the extension of it to gene-environment interaction by Ma et al. (2012) entail a so-called functional formulation. Whereas the statistical formulation is population-referenced and thus its parameters reflect properties of populations, the functional formulation is individual-referenced and thus its parameters reflect plane effects of substitutions from a reference class (a genotype at an environment) to the others. Applying that functional formulation from the default (non-exposed and non-risk) individual reference (0.01), the additive, dominance, environment, additive-by-environment, and dominance-by-environment effects reflecting the aforementioned substitutions are 0.245, 0.245, 0,39, 0.005, and 0.005, respectively (see Table 2). Those values show that, although the RAE case by Li et al. (2019) entails both genetic and environmental effects, it can hardly be considered a gene–environment interaction case as intended, since the gene–environment interaction effects are extremely small relative to both the genetic and the environmental marginal contributions—the interaction effects actually lie about two orders of magnitude below the marginal effects.

**TABLE 2:** Genetic/environmental effects of the two cases, RAE and RTE, considered in the text and detailed in Table 1.

		**Genetic/environmental effects**				
**Case**	**Reference**	**Additive**	**Dominance**	**Environment**	**AEI**	**DEI**
RAE	0.01	0.245	0.245	0.39	0.005	0.005
	0.245	0.393	0.246	0.394	0.008	0.005
RTE	0.01	0	0	0.39	0.25	0.25
	0.081	0.096	0.06	0.57	0.4	0.25

*AEI and DEI stand for additive–environment interaction and dominance–environment interaction, respectively. For each of the two cases, this table shows both functional (i.e., individual-referenced) effects from the reference of the default (non-risk and non-exposed class, with a disease susceptibility of 0.01) and statistical (i.e., population-referenced) effects from the reference of the average phenotype. The reference population of the statistical effects has a frequency of exposure of 0.24, a frequency of the risk allele of 0.15, Hardy–Weinberg proportions, and random associations of (i.e., absence of correlation between) genotypes and environments. By virtue of the latest, the results here reported may be equally obtained using the former NOIA setting and ARNOIA (ultimately from expression 4), as explained in the text.*

Hitherto, it has been shown that relatively recent implementations of the classical models not only enable the analysis of the RAE case built up by Li et al. (2019) to illustrate their theoretical proposals but are also adequate to easily and precisely quantify basic properties of that case itself, which have apparently been missed by those authors. More generally speaking, theoretical developments stemming from the classical models are not always fairly acknowledged. To this regard, it is worth noting that both NOIA (Álvarez-Castro and Carlborg, 2007) and the extension of it to gene-environment interaction by Ma et al. (2012) can properly deal with departures not only from complete dominance but also from Hardy–Weinberg proportions, which were assumed above only due to the absence of any explicit specifications of departures from those features.

Nevertheless, the general warning Li et al. (2019) post on the use of genetic models still holds—the current state-of-the-art implementations of classical models of genetic effects, whether unfairly acknowledged or not, still leave room for further improvement. Indeed, the original NOIA proposal fails to properly account for nonrandom associations of marginal genotypic frequencies (i.e., assumes linkage equilibrium between/among the loci involved) and Ma et al. (2012) inherit that limitation in regards to nonrandom associations between the marginal frequencies of genotypes and environments (i.e., gene–environment correlation). Thus, those association-pending models shall hereafter be referred to as the former NOIA setting. Incidentally, it is imperative to overcome that limitation both because correlations between/among marginal frequencies may occur in populations and because they are in any case likely to achieve significant levels in the actual samples used in real data analyses.

## Gene–Environment Interaction Disentangled From Gene–Environment Correlation

Opportunely, it is hereafter shown that the gaps of the former NOIA setting for gene–environment interaction can be bridged. Indeed, new mathematical developments for studying gene–environment interaction are provided below, in which gene–environment correlation is properly implemented. Since the resulting theoretical proposal bridges the aforementioned associations-pending gap, it shall be referred to as ARNOIA (associations-resolved NOIA).

### Theoretical Developments

A biallelic locus A (with alleles A_1_ and A_2_) and two environmental instances (E_1_ and E_2_) of an environmental variable E are initially considered. This setting leads to six possible classes—combinations of genotypes and environments—and thus to six phenotypic expectations (e.g., six values of disease susceptibility). Those values are gathered in the column-vector of genotypic values, **G** = (G_ijk_), where the subscripts indicate genotype A_j_A_k_ at environment E_i_.

The genotypic values can be expressed in terms of genetic effects by means of regression model


$$\text{G}={\text{N}}_{\mu }\,\mu +{\text{N}}_{e}\,\text{e}+{\text{N}}_{\alpha }\,\alpha +{\text{N}}_{\delta }\,\delta +{\text{N}}_{\alpha \,e}\,\alpha \,\text{e}+\delta \,\text{e},\text{(Expression 1)}$$

in which the explanatory variables are the mean phenotype μ, the environmental effect, **e** = υ_1_ = (*e*_1_, *e*_2_)^T^ (where T stands for the transpose operation), the genetic additive effect, α = υ_2_ = (α_1_, α_2_)^T^, the dominance effect, δ = υ_3_ = (*δ*_11_, *δ*_12_, *δ*_22_)^T^, and the additive-by-environment effect, α**e** = υ_4_ = (*αe*_11_, *αe*_12_, *αe*_21_, *αe*_22_)^T^, and the residual term is the dominance-by-environment effect, δ**e** = η_4_ = (*δe_*ijk*_*).

Let **1**^(m)^ be a column vector of length *m* with all its scalars equal to 1, **I**^(^*^*n*^*^)^ an identity matrix of dimension *n*, $\text{N = }{\left(\begin{array}{l} \begin{array}{ccc} 2 & 1 & 0 \end{array}\\ \begin{array}{ccc} 0 & 1 & 2 \end{array} \end{array}\right)}^{\text{T}}$, and ⊗ the Kronecker product. Then, the design matrices in expression (1) can be expressed as:


$${\text{N}}_{\mu }=1^{\left(6\right)},{\text{N}}_{e}={\text{N}}_{1}={\text{I}}^{\left(2\right)}⊗1^{\left(3\right)},{\text{N}}_{\alpha }={\text{N}}_{2}=1^{\left(2\right)}⊗\text{N},{\text{N}}_{\mu }=1^{\left(6\right)},{\text{N}}_{e}={\text{N}}_{1}={\text{I}}^{\left(2\right)}⊗1^{\left(3\right)},{\text{N}}_{\alpha }={\text{N}}_{2}=1^{\left(2\right)}⊗\text{N},$$

$${\text{N}}_{\delta }={\text{N}}_{3}=1^{\left(2\right)}⊗{\text{I}}^{\left(3\right)}\,\text{and}\,{\text{N}}_{\alpha e,}={\text{N}}_{4}={\text{I}}^{\left(2\right)}⊗\text{N}.\text{(expression 2)}$$

Regression (1) with design matrices (2) is meant to be solved sequentially, as follows. Let the population frequencies be *p*_*ijk*_ and let **P** be the diagonal matrix of those frequencies, **P** = diag(*p*_*ijk*_). Then, the mean phenotype is μ = Σ*p_*ijk*_ G_*ijk*_*, the mean-corrected vector of genotypic values is η_0_ = **G** – **1**^(6)^μ, and the expressions for the remaining explanatory variables and the residual term of regression (1) come from computing,


υl=H~l⁢ηl-1⁢and⁢ηl=Ml⁢ηl-1,l=1⁢to⁢ 4,(expression 3)

where ${\tilde{\text{H}}}_{l}={\left({\text{N}}_{l}^{\text{T}}\,{\text{PN}}_{l}\right)}^{-1}\,{\text{N}}_{l}^{\text{T}}\,\text{P}$ and ${\text{M}}_{l}\;=\;{\text{I}}^{\left(6\right)}\;-\;N_{l}{\tilde{H}}_{l}$.

With this, a theory of population-referenced (i.e., statistical, orthogonal) genetic/environmental effects that properly accounts for both gene–environment interaction and gene–environment correlation is provided, which shall hereafter be referred to as a correlationwise orthogonal interactions (COIA) model. In order to fully integrate COIA within the aforementioned NOIA framework (Álvarez-Castro and Carlborg, 2007), regression (1) has to be expressed in the form of a standardized statistical formulation. Such a formulation is


G=S⁢E,(expression 4)

where **E** = (*μ*, α, *δ*, *e*, *αe*, *δe*)^T^ is the vector of genetic/environmental effects and the genetic/environmental-effects design matrix, **S**, is computed via its inverse, **S**^–1^, whose rows can be obtained using expressions (1–3) where: the first one is (*p*_*ijk*_), the set of coefficients of *μ* = Σ*p_*ijk*_ G_*ijk*_*, the second, third, fourth and fifth ones are, analogously, the sets of coefficients of *G*_*ijkl*_ in α = (α_2_ – α_1_), *δ* = *δ*_12_ – ((*δ*_11_ + *δ*_22_)/2), *e* = *e*_2_ – *e*_1_ and *αe* = (*αe*_11_ – *αe*_12_ – *αe*_21_ + *αe*_22_), respectively, and the sixth one is (½, −1, ½, −1/2, 1, −1/2).

From expression (4) it is easy to perceive how critical building fine-tuned genetic/environmental-effects design matrices becomes in order to perfectly grease the machinery of the NOIA model, (here upgrading it in particular to an ARNOIA level, i.e., resolving the implementation of any kind of associations by means of COIA). Indeed, expression (4) is a compact way of representing how a genotype-to-phenotype map (essentially, a **G** vector) can be translated into its evolutionary properties. As noted by Álvarez-Castro and Carlborg (2007), the statistical, orthogonal genetic (and, here, also environmental) effects reflecting evolutionary properties of two populations, “1” and “2,” can easily be transformed into each other by equating the genotypic values in expression (4), i.e., simply as E_2_ = (S_2_)^–1^S_1_E_1_. And in what regards the individual-referenced (i.e., functional, natural) side of NOIA, this expression holds when one of the vectors of genetic/environmental effects (or both) and its corresponding matrix do not reflect allele substitutions made from the reference of a population, but of an individual genotype/environment instead. As pointed out above, functional (natural) genetic/environmental effects design matrices for gene–environment interactions imply a biallelic locus and two environments have been provided by Ma et al. (2012).

Using previous extensions of classical models of genetic effects (Álvarez-Castro and Yang, 2011; Alvarez-Castro and Crujeiras, 2019), the COIA regression framework for gene–environment interaction developed above and its implementation into an ARNOIA model can be extended to several, possibly multiallelic, loci with arbitrary epistasis and arbitrary departures from linkage equilibrium and simultaneously to several environmental variables with multiple environmental instances, with nonrandom associations (i.e., correlations) of environmental variables and of genotypes and environments. The details of such extensions are, though, beyond the scope of this paper.

### How Much of an Improvement?

**FIGURE 1:**
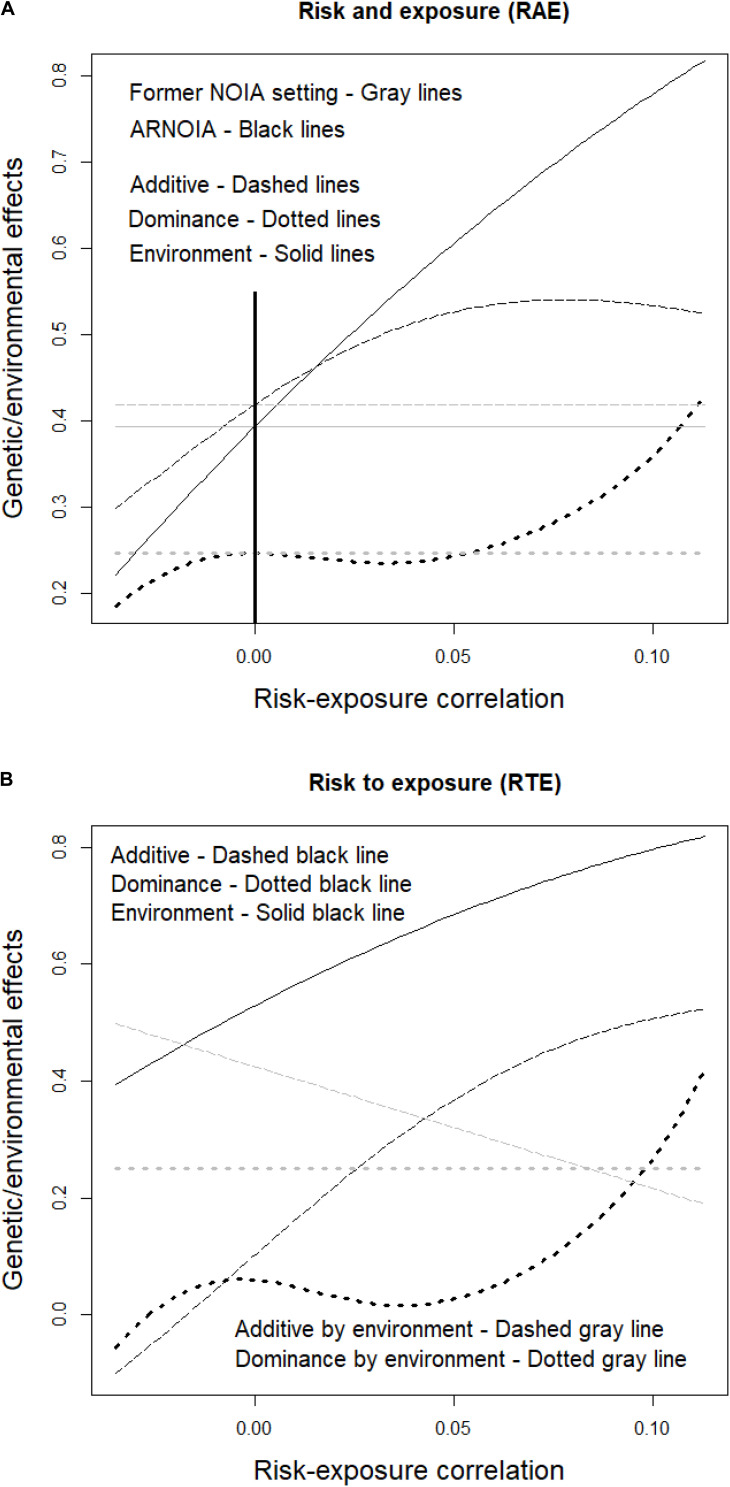
Genetic and environmental effects of disease susceptibility influenced by a risk allele and environmental exposure, for the whole range of possible correlations (including negative and positive associations) of the risk allele and environmental exposure. The risk allele frequency is 0.15, with genotypic frequencies under Hardy–Weinberg equilibrium, and the environmental exposure frequency is 0.24. The RAE case (see Table 1) is shown in panel **(A)**, where the former NOIA setting and ARNOIA, shown with gray and black lines, respectively, are compared. The thick black solid vertical line marks the case of random association (i.e., no correlation) between risk and exposure. The genetic effects obtained with ARNOIA for the RTE case (see Table 1) are shown in panel **(B)**. The marginal genetic and environmental effects are shown with the same black lines as in panel **(A)** and gray lines are used here for the interaction effects.

The advantage ARNOIA confers over the shoulders it stands on—the ones of the former NOIA setting (Álvarez-Castro and Carlborg, 2007) and, particularly, of its implementation with gene–environment interaction (Ma et al., 2012)—is discussed hereafter. As a baseline, the population-referenced genetic/environmental effects of the RAE case in the absence of gene–environment correlation are shown in Table 2 and they can be equally computed using either of the two methods. The whole range of possible correlations between the risk allele and environmental exposure is inspected in Figure 1A. The thick vertical line marks the point of random association (i.e., no correlation) where all values provided by the former NOIA setting by Ma et al. (2012) are correct and meet the ones provided by ARNOIA (i.e., as mentioned right above, the values in Table 2). Then, the model from Ma et al. (2012) still provides, within the whole range of correlations, those same values shown in Table 2 to fit to the random association scenario (gray horizontal lines) whereas ARNOIA (black lines) shows instead how the genetic/environmental effects actually change with negative (to the left of zero) and positive (to the right) risk-exposure correlations. Roughly, the effects decrease and increase with negative and positive correlations, respectively, although a slight decrease of the additive effect toward the maximum positive correlations and slightly more capricious behavior of the dominance effect for intermediate positive correlations can also be noticed.

In view of Figure 1A, it could seem that settling for the relatively simpler formulae of the former NOIA setting (not accounting for nonrandom associations of genes and environments) by Ma et al. (2012) would not come with a high cost. Indeed, values that are correct for circumstances known beforehand (precisely, nonrandom associations) are retrieved regardless the nonrandom associations involved. However, that is but a mirage for such a constraint shall, on the one hand, severely hamper the flexibility of the model for making predictions (as illustrated in the following section) and, on the other hand, make the models less efficient in disclosing genetic architectures (as explained below).

In what follows, a case of actual gene–environment interaction is considered. It is a case of genetic risk to environmental exposure (thus referred to hereafter as RTE), where the risk allele increases disease susceptibility only when combined with exposure, hence actually interacting with the environment. Thus described, the interaction behaves as a switch—the environmental effect shall either be switched on (when carrying the risk allele) or turned off (otherwise), as shown in Table 1.

Table 2 shows that the functional additive and dominance effects (i.e., the marginal genetic effects) of the RTE case from the reference of the individual default class (no genetic risk and no exposure) are zero, which is in accordance with the genetic risk being turned off in the absence of exposure. In Table 2 it is also illustrated that large gene-interaction effects actually have a noticeable influence on the lower level effects, since marginal genetic effects become not nil under a different genetic/environmental background (i.e., when expressing the effects from a different reference) and also the environmental effect is significantly modified. In the RAE case, only the additive effects change noticeably under different references, which is an effect of dominance interaction under backgrounds with differential presence of the alleles.

For a broader scope, Figure 1B shows all the genetic/environmental effects of the system as obtained using ARNOIA, for the whole range of possible gene–environment correlations. Marginal effects are displayed as in Figure 1A and gene–environment interaction effects are shown in gray. The marginal genetic effects of the RTE case are small in the absence of gene-environment correlation. Indeed, this case entails a visual example of a warning issued above since it illustrates that marginal effects approach zero as an occasional outcome (of a particular set of population frequencies), making it tricky to spot them in a mapping experiment. The trouble vanishes though as long as the (larger) gene–environment interaction effects are inspected (despite the apparent absence of marginal genetic effects) and disclosed. Note also that although the marginal genetic effects get closer to zero under certain negative correlations (toward the far-left end of the graph), the additive-by-environment interaction effect increases accordingly. Thus, in any case, eventually out-of-reach marginal effects may be unveiled by diligently fishing interaction effects.

Overall, for properly detecting marginal (genetic and environmental) and interaction (gene–gene and gene–environment) effects (and, therefore, identify their corresponding loci and environmental variables) in mapping experiments it is essential that the genetic models entail not only any interactions between/among the effects themselves but also any departures from equilibrium genotype/environment frequencies, as Figure 1 shows ARNOIA to accomplish. Moreover, it is hereafter illustrated that the advantages of ARNOIA over the former NOIA setting are also crucial for using detected genetic and environment underlying factors of traits in the formulation of predictions, particularly in the context of precision medicine.

### Predictions Under Diminishing Exposure

**FIGURE 2:**
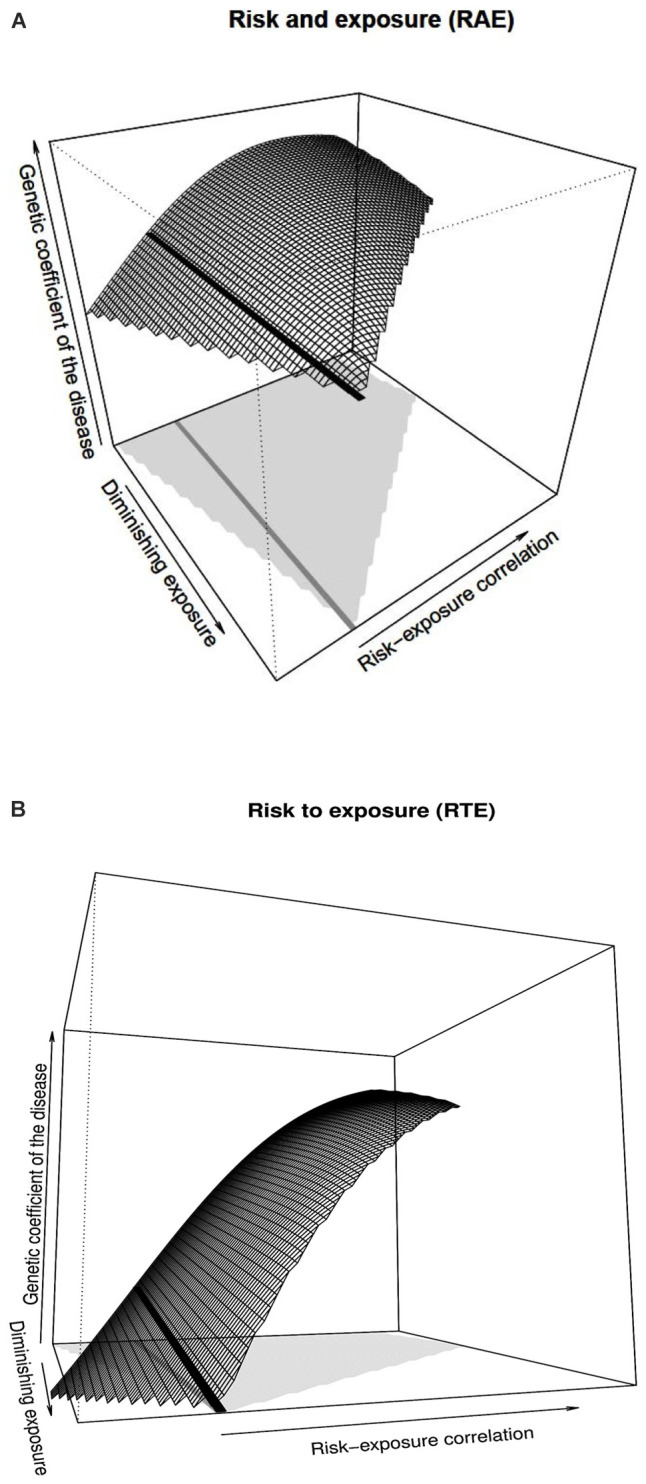
Genetic coefficient of the disease obtained with ARNOIA for the RAE and RTE cases, shown in panels **(A)** and **(B)**, respectively. The details of panels **(A)** and **(B)** of Figure 1 apply to panels **(A)** and **(B)** of Figure 2, respectively. The range of possible risk-exposure correlations is shown by the light gray area at the bottom of each graph. The values of the vertical axis range from 0 to 1.20. The thick black line (whose projection at the bottom of the figure is shown in dark gray) marks the absence of correlation between risk allele and environmental exposure, which are the ones the former NOIA setting would provide for the whole range of correlations between the risk allele and environmental exposure.

Figure 2A shows the genetic coefficient of the disease—as defined by Li et al. (2019)—for the RAE case, which, as mentioned above, in the context of the developments stemming from the classical models of genetic effects is given by the parameter 2α. On top of the variables already considered in Figure 1A, Figure 2A has one dimension added for enabling predictions in the context of a hypothetical decrease of the environmental exposure. The black solid line in Figure 2A marks random association and shows that the genetic coefficient of the disease is simply not affected by decreasing the exposure frequency in the population. This is as expected under the lack of interplay between gene and environment (i.e., no interaction and no correlation). Indeed, although the trait is subject to both genetic and environmental influence, as long as there is no (or very little) interplay between them, the genetic parameter remains virtually constant in the face of variations in the environmental exposure.

However, as already shown above in relation with Figure 1 (where the additive genetic effect, α, was shown instead of the genetic coefficient of the disease, 2α), such an interplay may come not only by means of gene–environment interaction but also through gene–environment correlation. Thus, whereas the genetic coefficient of the disease remains constant in Figure 2A against diminishing exposure in the absence of significant gene–environment interaction, it is in point of fact affected by risk-exposure correlations. In particular, negative associations between the risk allele and exposure causes the genetic coefficient of the disease to decrease, as the surface to the left of the black line shows. Conversely, positive associations make it increase, to the right of the black line, although this occurs up to a maximum followed by a slight decrease. This is, naturally, the same kind of behavior the additive effect, α, displays in Figure 1A. Note also that the range of possible risk-exposure correlations (shown by the light gray area at the bottom of the figure) narrows down as the exposure frequency approaches zero, which explains the tip of the surface at the end of the black line.

In Figure 2B, the RTE case of Figure 1B is resumed and further extended in a way analogous to Figure 2A from Figure 1A. As Figure 2B shows, for the RTE case the genetic coefficient of the disease decreases for decreasing values of exposure under random associations of risk and environment (decreasing black line). That coefficient also decreases for decreasing (increasingly negative) associations between the risk allele and environmental exposure, as the left tip of the surface shows. In plain language, the figure shows that the problem of increased disease susceptibility of the carriers of the risk allele may be reduced (and eventually removed) either by reducing exposure for the whole population or by restricting the access to the exposed environment only for the risk population, or even through any intermediate alternative (any reduction of the exposure in the population biased toward the carriers of risk alleles). Optimal management would then depend just upon the reluctance of the average individual to avoid the exposed environment (or even the actual feasibility of bringing the whole population out of it) and the cost of tests to detect the risk allele, which would enable personalized warnings.

Overall, the RAE and RTE cases considered in Figure 2 deal with rather singular instances (virtually absent and switch-type, respectively) of gene–environment interaction, for which some predictions would be feasible even without mathematical modeling. The results obtained using ARNOIA not only reassuringly agree with the conceptually attainable predictions but also further illustrate how to precisely quantify any desired genetic/environmental parameter. Such an advantage can hereafter be applied to more complex real cases of interest undergoing less intuitive behaviors.

## Discussion

### An Accurate Route Planner for Complex Genetic Architectures

Interactions are known to encrypt the map where a pursued genetic architecture could be spotted. This is known to occur because interactions of any kind (from just dominance to gene–environment interaction) may make lower level effects (like environmental effects or genetic additive effects) vanish under a certain genetic/environmental composition of a population or of an experimental sample (see e.g., Álvarez-Castro, 2012). This fact, which has been further illustrated in Figure 1A, is unfortunately not always properly taken into account. In relation with this, the commendable review by Malosetti et al. (2013) on models of gene–environment interaction in the context of plant breeding reasonably recommend to adhere to a strategy where effects are inspected sequentially—as they also are in expressions (1–3) above—, but it oversteps the mark when more specifically proposing a conditional sequential procedure, by claiming that “dominance effects should be tested conditioned on the additive effects present in the model.” Indeed, since interactions make effects on the phenotype able to cancel out in average at the group of individuals under study and to thus be missed in mapping experiments, unveiling interactions actually becomes doubly imperative rather than something to be subject to the condition of first having found their (possibly masked) lower-order effects.

Thus, in order for the theoretical genetic/environment models and the estimation strategies used in mapping methods to become accurate enough to address the difficulty of dealing with possibly masked effects, it is necessary in the first place to opportunely implement such models with interaction effects, as thoroughly recalled by Li et al. (2019), particularly in regards to precision medicine. But interactions are deceitfully puzzling and thus trying to properly map complex genetic architectures makes it crucial to also improve the flexibility of the models to accurately fit to the frequencies of the genotypes in the population/sample under study. Indeed, Zan et al. (2018) have recently shown to what extent departures from linkage equilibrium frequencies may condition the models to strikingly distort the genetic architecture of a trait, particularly in regards to genetic interactions. To this regard, it has become particularly opportune that an elusive implementation of models of genetic interactions, even claimed to be beyond reach, has recently been attained, enabling genetic interaction (epistasis) and genotype frequencies correlation (linkage disequilibrium) to be disentangled (Alvarez-Castro and Crujeiras, 2019). The COIA regression framework and the ARNOIA model developed above attain an analogous goal in what regards a joint implementation of gene–environment interaction and gene–environment correlation, which is particularly timely for aiding precision medicine, as further discussed below.

### The Road Maintenance System

Affordable data is an ever-changing variable and it is thus sensible to assume that, likewise, theoretical models required in data analyses shall need to keep on being worked out every now and then. In this context, it is as essential to make the best possible use of the models available at a particular time-spot as it is to point out in which precise way they are at that time imposing limitations on the analyses. In what has been recently understood as the, at least relative, “failure of GWAS” (Teperino, 2020b), gene–environment interaction has been pointed out as a key factor. Indeed, the importance of gene–environment interaction in human health has been stressed in relation with a broad spectrum of disorders ranging from obesity, cardio-metabolic diseases, and other metabolic disorders, through to cancer, autoimmune diseases, and mental disorders (e.g., Karl and Arnold, 2015; Lopizzo et al., 2015; Flouris et al., 2017; Cust, 2020; Smith, 2020; Teperino, 2020a). All in all, the lack of a fine-tuned theory of gene–environment interaction has, thus, been imposing serious limitations in this field.

More precisely, developments implementing the effects of both gene–environment interaction and gene–environment correlation in a properly disentangled manner have explicitly been demanded within the field of precision medicine. Indeed, in the context of mental health, Assary et al. (2020) have recently advocated that “[i]dentifying which form of gene–environment interplay contributes to a particular disorder or behavior is absolutely crucial in order to select suitable intervention efforts” because theoretical developments that enable a joint analysis of both phenomena are needed, in particular for “ensuring that the outcomes of one do not bias the effects of the other.” The present paper meets that demand and it does so within a theoretical framework capable of simultaneously addressing many other genetic facts of relevance.

Indeed, the ARNOIA model here provided illustrates the possibilities of mathematical developments stemming from the classical models of genetic effects in regards to their potencial to be continuousy improved and thus address eventual demands to come. In other words, since the machinery here provided has proven useful to fill in inconvenient potholes of the classical road network, it should be deemed to be applicable to increasingly complex challenges in the future. For instance, the need to consider gene–gene–environment interaction (e.g., Zan and Carlborg, 2020) and/or gene–environment–environment interaction (e.g., Keers and Pluess, 2017) has already arisen and it is to this regard worth highlighting here that the advantages of ARNOIA can also be applied to address such complexities (and actually gene–environment interaction/correlation, multiple alleles, dominance, epistasis and departures from Hardy-Weinberg equilibrium and from linkage equilibrium, simultaneously) by merging the mathematical developments provided above with previous theory (Álvarez-Castro and Yang, 2011; Alvarez-Castro and Crujeiras, 2019).

The previous is, however, not to say that alternative roads—like Li et al. (2019)—should never be built. It looks sensible in any case to assume that a new road will consume significant resources before providing benefits comparable to the already existing ones, especially in regards to the wealth of experience amassed in the use of them. Therefore, it would be reasonable to first thoroughly inspect the possibilities of the existing roads to be fixed and as well to guarantee the added value the new road is intended to bring. On top of that, it would also make perfect sense to assume that the new road would only provide its best service when adequately connected with the previous road network. Whenever developed along these lines, alternative perspectives in genetic modeling could aim to open doors to novel analyses and/or double check the already existing ones and thus enrich the application of mathematical models of genetic and environmental effects in precision medicine.

### Accurately Assessing COVID-19

As a final remark, it would be regrettable in the context of the current COVID-19 pandemic not to explicitly point out that ARNOIA improves the methodology that can in particular be applied to dissect the behavior, and thus help to eventually overtake, such a global threat. Epidemiology relies on a thorough study of interactions (see e.g., Dewan, 2018) and the particularly strong link between epidemiology and gene–environment interaction has already been underscored in relation to the COVID-19 pandemic (Rodriguez-Morales et al., 2020). In the cases analyzed above, ARNOIA has been applied to disease susceptibility and from those instances it becomes easy to perceive that it is equally applicable to other traits of interest in epidemiology, including, for instance, mortality caused by a disease.

Generally, the dynamics of a pandemic shall depend upon how the different strains of the infectious agent affect different (groups of) individuals, with different (proportions of) genotypes and under different environmental conditions. It becomes particularly useful to notice in this regard that although virulence variability is underlain by mutations (and thus conceptually related to genetics), ARNOIA may naturally integrate the presence of different strains (with differential virulence) simply as an (additional) environmental variable, since that is how they are perceived from the perspective of the susceptible individuals—the genetic component of the model. Bearing that in mind, it is easier to perceive why it is crucial, for the study of COVID-19, that ARNOIA considers together (but disentangled) gene–environment interaction and gene–environment correlation. Indeed, the various geographical regions affected by the disease do not only undergo different proportions of virus strains (environmental component of the model) but also different genetic backgrounds of the susceptible individuals (genetic component), thus setting a human genotype–strain (gene–environment) correlation scenario in which human genotype–strain (gene–environment) interaction needs to be properly understood.

## Data Availability Statement

The original contributions presented in the study are included in the article/supplementary material, further inquiries can be directed to the corresponding author.

## Author Contributions

The author confirms being the sole contributor of this work and has approved it for publication.

## Conflict of Interest

The author declares that the research was conducted in the absence of any commercial or financial relationships that could be construed as a potential conflict of interest.
