# Corrigendum: Gene-environment interaction in the era of precision medicine—filling the potholes rather than starting to build a new road

**DOI:** 10.3389/fgene.2023.1231652

**Published:** 2023-06-21

**Authors:** José M. Álvarez-Castro

**Affiliations:** Department of Education, Xunta de Galicia, University and Professional Training, Santiago de Compostela, Spain

**Keywords:** gene–environment interaction, gene–environment correlation, precision medicine, disease susceptibility, COVID-19, mathematical model, NOIA

In the published article, there was an error in Figure 2B as published. The surface shown in the figure, although similar to the correct one, is wrong—it does not clearly take negative values at the left of the panel, as it should. The corrected Figure 2B appears below.

**FIGURE 2B:**
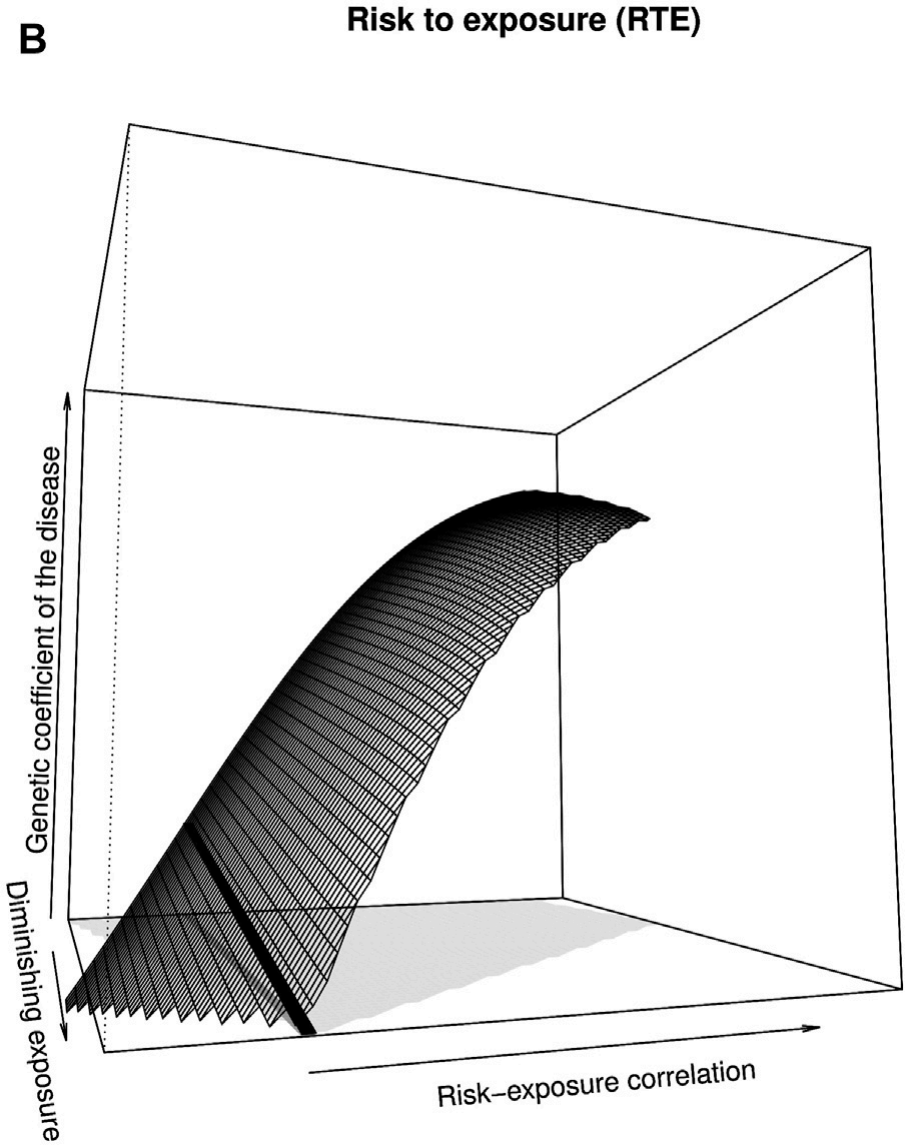


The authors apologize for this error and state that this does not change the scientific conclusions of the article in any way. The original article has been updated.

